# Opposing CSF hydrodynamic trends found in the cerebral aqueduct and prepontine cistern following shunt treatment in patients with normal pressure hydrocephalus

**DOI:** 10.1186/s12987-019-0122-0

**Published:** 2019-01-22

**Authors:** Robert B. Hamilton, Fabien Scalzo, Kevin Baldwin, Amber Dorn, Paul Vespa, Xiao Hu, Marvin Bergsneider

**Affiliations:** 1 Neural Systems and Dynamics Laboratory, Department of Neurosurgery, The David Geffen School of Medicine, University of California-Los Angeles, 10833 Le Conte Ave, Los Angeles, CA 90095 USA; 2 Biomedical Engineering Graduate Program, Henry Samueli School of Engineering and Applied Science, University of California-Los Angeles, 7400 Boelter Hall, Los Angeles, CA 90095 USA; 3 The David Geffen School of Medicine, University of California-Los Angeles, 10833 Le Conte Ave, Los Angeles, CA 90095 USA; 4 Neural Analytics, Inc., 2440 S Sepulveda Blvd, Suite 115, Los Angeles, CA 90064 USA

**Keywords:** Phase contrast MRI (PC-MRI), Normal pressure hydrocephalus, Aqueduct of Sylvius, Cerebrospinal fluid (CSF), Prepontine CISTERN, Cerebral compliance

## Abstract

**Background:**

This study investigated cerebrospinal fluid (CSF) hydrodynamics using cine phase-contrast MRI in the cerebral aqueduct and the prepontine cistern between three distinct groups: pre-shunt normal pressure hydrocephalus (NPH) patients, post-shunt NPH patients, and controls. We hypothesized that the hyperdynamic flow of CSF through the cerebral aqueduct seen in NPH patients was due to a reduction in cisternal CSF volume buffering. Both hydrodynamic (velocity, flow, stroke volume) and peak flow latency (PFL) parameters were investigated.

**Methods:**

Scans were conducted on 30 pre-treatment patients ranging in age from 58 to 88 years along with an additional 12 controls. Twelve patients also received scans following either ventriculoatrial (VA) or ventriculoperitoneal (VP) shunt treatment (9 VP, 3 VA), ranging in age from 74 to 89 years with a mean follow up time of 6 months.

**Results:**

Significant differences in area, velocity, flow, and stroke volume for the cerebral aqueduct were found between the pre-treatment NPH group and the healthy controls. Shunting caused a significant decrease in both caudal and cranial mean flow and stroke volume in the cerebral aqueduct. No significant changes were found in the prepontine cistern between the pre-treatment group and healthy controls. For the PFL, no significant differences were seen in the cerebral aqueduct between any of the three groups; however, the prepontine cistern PFL was significantly decreased in the pre-treatment NPH group when compared to the control group.

**Conclusions:**

Although several studies have quantified the changes in aqueductal flow between hydrocephalic groups and controls, few studies have investigated prepontine cistern flow. Our study was the first to investigate both regions in the same patients for NPH pre- and post- treatment. Following shunt treatment, the aqueductal CSF metrics decreased toward control values, while the prepontine cistern metrics trended up (not significantly) from the normal values established in this study. The opposing trend of the two locations suggests a redistribution of CSF pulsatility in NPH patients. Furthermore, the significantly decreased latency of the prepontine cisternal CSF flow suggests additional evidence for CSF pulsatility dysfunction.

## Background

The pathophysiology of normal pressure hydrocephalus (NPH) and that of communicating hydrocephalus in general, remains an enigma. The traditional tenet, dating back more than 70 years to the work of Walter Dandy [1], posited that cerebrospinal fluid (CSF) malabsorption or obstruction at the level of the arachnoid granulations was responsible for the accumulation of CSF within the ventricles. Several lines of evidence suggest that alternative mechanisms may be at play [2–5], including the hypothesis that interference in pulsatile CSF dynamics plays an important role [6, 7], and that absorption may take place into the parenchymal capillaries [8]. Williams proposed that venous dysfunction is an important initiating factor in the etiology of NPH [9]. We previously proposed [6] that a possible underlying mechanism relates to the role of pulsatile CSF inflow and outflow across the foramen magnum in relation to changes in cerebral blood volume with every heartbeat. Rather than relegating the rapid caudal flow of CSF seen on cine MRI flow studies to a response to the net increase in arterial cerebral blood volume during systole, we proposed that cranial-spinal CSF volume buffering is intimately related to cerebral blood flow reactivity (the response of cerebral blood flow to changes in vasculature analogous to an increase in induction). The term buffering refers to the ability of the CSF to dampen the arterial input into the cranial vault (the amplitude of the arterial pulse). Arterial resistance is the amount of wall resistance that the blood must overcome in order to flow through that particular vessel. A reduction in CSF volume buffering would increase CBF reactivity leading to a compensatory reduction in arterial resistance to maintain a constant CSF pressure.

Greitz et al. [10] elegantly demonstrated that pulsatile CSF movement occurs via two basic routes: the cisterns and ventricles. The larger cisternal movement occurs in response to an up-and-down piston-like motion of the brain with every heartbeat driven in relation to changes in blood volume in the subarachnoid spaces. The smaller CSF movement out and back into the ventricles, on the other hand, resulted from a medial (normal to the surface of the brain) movement of the cerebral hemispheres, primarily as a result of an increase in brain volume due to the increase in blood volume. Both cisternal and ventricular CSF volume buffering are important for normal cerebral hemodynamics.

Here, we hypothesized that communicating hydrocephalus (CH) arises primarily from a disturbance of either cisternal or other subarachnoid CSF pulsatile movement. In some respects, this concept is not too different from the Dandy traditional theory except that the putative point of “obstruction” to CSF flow is “upstream” from the arachnoid granulations and related primarily to pulsatile movement rather than solely on bulk CSF flow. Egnor et al. published a model on CH that suggested the increase in ventricular pulsatility (causing ventriculomegaly) was a result of increased impedance in the subarachnoid space (SAS) [11]. This idea did not develop exclusively based on theoretical machinations, but rather as a result of trying to explain a well-established observation in NPH: that the CSF stroke volume (SV) through the cerebral aqueduct of Sylvius is markedly elevated in NPH [12–14]. We reasoned that a reduction in cisternal and/or subarachnoid CSF volume buffering would have to be compensated by an increase in ventricular buffering—thereby producing aberration in pulsatile CSF dynamics described by Bradley [15] and others [16–18].

CSF pressure and flow oscillations within the cranium originate from the arterial pulsations, causing changes in cerebral blood volume entering the cranial vault through the internal carotid and vertebral arteries [10]. CSF flow from ventricle and intracranial subarachnoid spaces into the spinal compartments comprise the majority of the bulk flow [19]. Phase contrast (PC)-MRI has measured increased amplitudes of fluid flow through the aqueduct during the cardiac cycle [7]. It has been shown by Wagshul et al. [20] and others [10, 17, 19, 21] that the CSF latency (temporal difference in peak flow in relation to the cardiac cycle) varies throughout the cranial vault. Additionally, it has recently been established that some attributes of net CSF flow even vary with different phases of the respiratory cycle [22]. Ventricular CSF flow represents a very small but important part of the system; it is where the spinal CSF flow originates [19]. Using PC-MRI and the carotid arteries as a reference, Wagshul et al. showed a shorter latency in the prepontine cistern pulse compared with the cerebral aqueduct pulse [20]. Combining this information with aspects of Egnor’s model of CH [11] we could also reasonably expect alterations in CSF flow latency between the cerebral aqueduct and the prepontine cistern. In fact, it has been well-established that hyperdynamic aqueductal CSF oscillations are found in NPH patients [23]. Specifically, we hypothesized that pre-treatment NPH patients should have shorter latency in both the cerebral aqueduct and prepontine cistern.

The treatment of CH typically entails the implantation of a CSF “shunt,” a diversionary system that allows CSF flow through a catheter from the ventricle to either the peritoneum or atrium of the heart. The success of these shunting procedures can be variable and is dependent on patient selection and timing of procedure [24]. In terms of CSF pulsatile dynamics, a CSF shunt offers an alternative pathway for CSF volume buffering. We therefore further hypothesized that successful implantation of a CSF shunt in a hydrocephalic patient would result in normalization of both the aqueductal and cisternal SVs towards control values.

## Methods

### Study cohort and image acquisition

This study measured CSF flow in the cerebral aqueduct and the prepontine cistern using PC-MRI in three distinct groups: pre-shunt NPH patients, post-shunt NPH patients, and controls. All imaging and procedures were approved by the IRB committee and patients and normal controls provided written consent prior to the imaging (10-001128, 06-11-013, and 07-08-038). Scans were conducted on 30 pre-treatment patients (77.8 ± 7.1 year, 19 males and 11 females) ranging in age from 58 to 88 year and 12 controls (66.3 ± 9.2 year, seven males and five females). Additionally, 12 patients received scans following either ventriculoatrial (VA) or ventriculoperitoneal (VP) shunt treatment (nine VP, three VA), ranging in age from 74 to 89 year (81.7 ± 4.6 year) with a mean follow up time of 6 months, the remaining patients either received an endoscopic third ventriculostomy (ETV) or were not recommended for treatment. Of the 12 follow up scans, there were nine matched pre-post aqueduct scans and six matched cisternal scans.

All MRI scans were performed using a 3T Siemens Trio T-class MRI (Siemens Medical Systems, Erlanger, Germany). The participants were placed in the supine position with neck and head in the neutral position using a Siemens Head Matrix coil. All participants received the same imaging protocol, starting with anatomical sequences: a 3D axial T1-weighted MPRage gradient-echo sequence (1900 ms/3.44 ms/0.84375 mm/0.899 mm/320 mm × 320 mm/268.8 mm × 268.8 mm/9°, TR/TE/real acquired spatial resolution/slice thickness/matrix/FOV./flip angle), axial T2-weighted BLADE (7110 ms/107 ms/0.5729 mm/3 mm/384 mm × 384 mm/268.8 mm × 268.8 mm/120°), and a sagittal T2-weighted Turbo spin echo sequence (750 ms/100 ms/0.34375 mm/8 mm/616 mm × 640 mm/209.44 mm × 217.6 mm/170°).

**Fig. 1:**
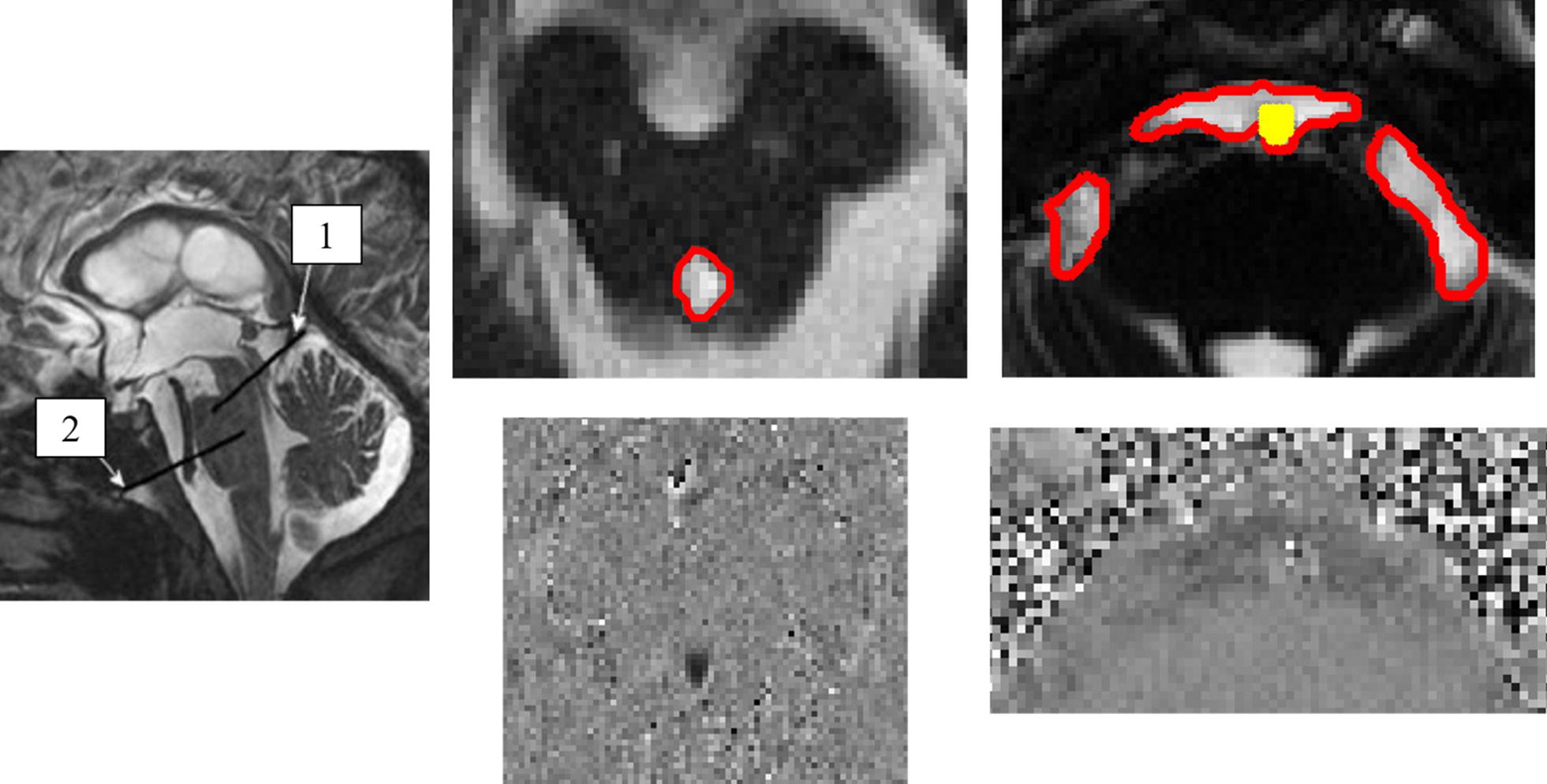
Left) Midsagittal T2-weighted image, flow acquisition planes for (1) cerebral aqueduct and (2) prepontine cistern. Planes were defined perpendicular to CSF flow. Center top) Example of cerebral aqueduct (T2 TruFisp) with the region of interest for the flow quantification outlined in red. Center Bottom) Example of the phase contrast sequence for the cerebral aqueduct during peak caudal CSF flow. Right top) Example of prepontine cistern (T2 TruFisp) with the region of interest for the flow quantification outlined in red and the basilar artery highlighted in yellow. Right bottom) Example of the phase contrast sequence for the prepontine cistern during peak caudal CSF flow

Flow quantification was achieved using a series of imaging sequences including localization, anatomical, velocity estimation, and phase contrast (PC). Using a midsagittal slice, an oblique plane was defined perpendicular to the presumed direction of CSF flow for both the aqueduct and prepontine cistern (Fig. 1). A true FISP (5.36 ms/2.36 ms/0.625 mm/3 mm/256 mm × 256 mm in aqueduct, 320 mm × 320 mm in cistern/299.68 mm × 199.68 mm in aqueduct, 200 mm × 200 mm in cistern/60°) steady-state coherent sequence was used to visualize the local anatomy of the oblique slice; CSF appears as hyperintense as contrast is determined by T2*. The velocity encoding parameter (V_enc_) is a variable set by the MRI technician and defines the range of the measured velocities in the phase contrast sequence. A flow scout sequence was used initially to estimate the range of V_enc_ values prior to setting the final V_enc_ for the phase contrast sequence which varied based on the peak flow velocity of each patient. Following the definition of the V_enc_, the phase contrast sequence (39.1 ms/6.01 ms/0.625 mm/3 mm/240 mm × 320 mm in aqueduct, 192 mm × 256 mm in cistern/150 mm × 200 mm in aqueduct, 149.7 mm × 199.68 mm in cistern/15°) was applied; to ensure its accuracy, the results were checked for aliasing and further adjustments to the V_enc_ were made, if necessary. For the aqueduct, the mean and standard deviation for the V_enc_ used was 17.8 ± 4.5 in the pre-shunt group, and 13.1 ± 4.9 for the post-shunt group. For the prepontine cistern pre-shunt group, the V_enc_ was 9.7 ± 5.39, and 7.6 ± 4.0 for the post-shunt group. The duration time of one PC-MRI acquisition was between 1.5 and 3 min for a single acquisition based on the period of the cardiac cycle. The cistern pre- and post- groups average beats per minute (BPM) were 66.9 ± 8.83 and 65.23 ± 11.59, respectively. The aqueductal pre-shunt and control groups had BPM 69.2 ± 8.3 and 66.6 ± 9.3, respectively. Finally for the PC-MRI sequence, there was retrospective gating with either ECG or pulse oximetry with a temporal resolution of 30 frames. Due to additional noise from arterial blood flow (basilar artery) in the phase contrast images of the prepontine cistern, a Time-of-Flight sequence (24 ms/3.69 ms/0.78 mm/0.8 mm/216 mm × 320 mm/168.4 mm × 249.6 mm/18°) aided the segmentation from the phase contrast sequence.

### Data analysis

A semi-automated segmentation algorithm was implemented for the designation of the region of interest (ROI) for the cerebral aqueduct and the prepontine cistern. The algorithm utilized dynamic time series information coupled with spatial information [25] for segmentation of the individual voxels used in the analysis. The segmentation algorithm was developed using MATLAB 7.5 R2007b (The MathWorks, Inc., Natick, MA, USA) with a general description below [26].

#### Algorithm overview

The algorithm used a three-step process: (i) reference waveform generation, (ii) correlation map construction, and (iii) threshold determination. First, an edge detection algorithm segments the CSF from the magnitude image provided by the phase contrast sequence. The selected voxels from the binary mask are used to create the reference waveform by aggregating time-series information from the 30 frames of the PC-MRI sequence. Next, dynamic (temporal) information is included into the segmentation algorithm by comparing the reference waveform to each voxel’s time-series information and builds a correlation map. The dynamic information improves in the segmentation of regions impacted by flow voids. Finally, a threshold value is used to segment the final region [26].

#### CSF dynamics quantification

Following the determination of the ROI, several CSF hydrodynamic metrics are derived from the PC-MRI sequence: velocity, flow, and stroke volume. Prior to calculating these metrics, a phase correction was made to offset accumulated phase or eddy currents due in part to the position of the patient in the scanner and the intrinsic properties of the magnet by selecting a region within the midbrain, and subtracting the average velocity over the 30 timepoints from the ROIs in the cerebral aqueduct and pre-pontine cistern, this methods has previously been described [19]. The velocity (cm/s) is derived from the intensity waveforms obtained from the PC-MRI sequence after correction of the V_enc_. The metrics included both maximum (peak) and mean velocity for both the caudal and cranial directions. Flow (mL/min) was computed on a voxel-by-voxel basis, by incorporating the pixel area and integrating over the ROI. Bradley et al. defined aqueductal stroke volume (SV) as the average of the volume of CSF moving in the cranio-caudal direction and the volume moving in the caudo-cranial direction [8]; this is in comparison to Bateman et al., which defined the SV as the area between the baseline (zero flow) and the peak portion of the flow curve [27]. In practice, these values should be approximately equal due to the near-zero bulk flow throughout the cardiac cycle; however, only values based on Bradley’s definition will be reported in this study. Finally, the ROI area was compared among the three groups for both the aqueduct and prepontine cistern.

#### Peak flow latency calculation

Peak Flow Latency (PFL) is defined as the percent cardiac cycle at peak caudal CSF flow in the cerebral aqueduct and prepontine cistern. Following the calculation of the ROI, several additional steps were needed to reliably calculate the PFL. First, due to influence of partial volume, the ROI boundary voxels were removed to increase flow signal. Second, the remaining voxel’s temporal waveforms were averaged to produce an intermediate reference waveform (this is the “characteristic flow” within the reduced ROI). The third step correlated the reference waveform to each with each voxel in the reduced ROI to rank representative flow velocity waveforms. Based on this value, the top 25% of highly correlated voxels were averaged to represent the final reference waveform (75% of the voxels were removed). The new reference waveform’s temporal resolution was limited to the PC-MRI imaging parameters which is 30 samples. Therefore, the final step in the calculation of the PFL was the fitting of a six degree polynomial to the final reference waveform which increased the temporal resolution from 30 to 1000 samples per cardiac cycle (selection of the six degree polynomial as well as the percentage of voxels used in the determination of the waveform are discussed later in the manuscript). The PFL latency was defined as the percent cardiac cycle at peak caudal CSF flow which is the minimum of this waveform. For the PFL to be comparable, only patients whose MRI was gated with ECG were used in this analysis.

#### Ventricle segmentation

For the nine patients that had pre- and post- treatment scans, the total lateral and third ventricle volumes were calculated (3DSlicer, http://www.slicer.org). The segmentation was performed semi-automatically, following the placement of a seed point in the lateral ventricles and then edited manually by an expert.

#### Patient outcome

Patient outcome was assessed at the time of the post-treatment scan, approximately 6 months following surgery. The outcome was based on clinical notes at the time of the clinic visit, with an emphasis on the improvement in gait based on the suggestion by Edwards et al. [28]. Although further valve adjustments were made for majority of patients, the outcome was assessed at the time of the post-treatment scan prior to any valve adjustment. All clinical evaluations were blinded to the results of the flow analysis presented in this work.

#### Statistical methods

All statistical analysis was performed using MATLAB 7.5 R2007b functions. For the comparison of the pre-treatment NPH and healthy control groups, the Mann–Whitney Rank sum test with a significant level of 0.05 was used. When comparing paired pre- and post-treatment NPH results the Wilcoxon signed rank test was used.

## Results

### Hydrodynamic results pre-shunt NPH and control group

Significant differences in area, velocity, flow, and aqueductal stroke volume (ASV) metrics for the cerebral aqueduct were found between the pre-treatment NPH group and the healthy controls. Mean flow and max velocity in both the caudal and cranial direction as well as ASV and ROI area were significantly higher in the pre-treatment group. Selected median and interquartile ranges are shown in Table 1.

**Table 1:** The quantitative results from the pre-shunt NPH and healthy control groups for both the aqueduct and prepontine cistern

	Area (mm^2^)	Caudal max velocity (cm/s)	Cranial max velocity (cm/s)	Caudal mean flow (mL/min)	Cranial mean flow (mL/min)	SV (μL)
Aqueduct						
Pre-shunt (n = 26)	8.0 [3.4]** (5.1–13.7)	12.8 [8.3]* (5.3–21.4)	8.4 [5.8]* (4.3–17.0)	0.32 [0.23]** (0.12–0.73)	0.26 [0.18]** (0.09–0.57)	124.5 [94.5]** (37.1–275.0)
Control (n = 10)	4.9 [1.7] (3.1–7.4)	7.9 [4.5] (5.3–15.0)	5.9 [2.2] (3.8–8.2)	0.12 [0.07] (0.06–0.19)	0.09 [0.04] (0.05–0.15)	49.7 [32.3] (23.1–90.5)
Cistern						
Pre-shunt (n = 21)	58.6 [63.8] (20.1–255.7)	6.3 [3.5] (2.7–14.1)	4.9 [3.5] (1.9–12.4)	0.78 [0.31] (0.3–2.0)	0.43 [0.31] (0.14–1.47)	293.5 [157.6] (111.9–683.6)
Control (n = 8)	69.6 [22.0] (31.1–92.2)	5.7 [1.8] (3.1–7.5)	4.3 [1.9] (2.1–8.3)	0.73 [0.35] (0.4–0.8)	0.41 [0.22] (0.27–0.63)	299.0 [171.5] (181.2–418.7)

Significant differences between NPH and control denoted by *(p < 0.05) and **(p < 0.001). For each metric the median [iqr] is shown. *SV* stroke volume

**Fig. 2:**
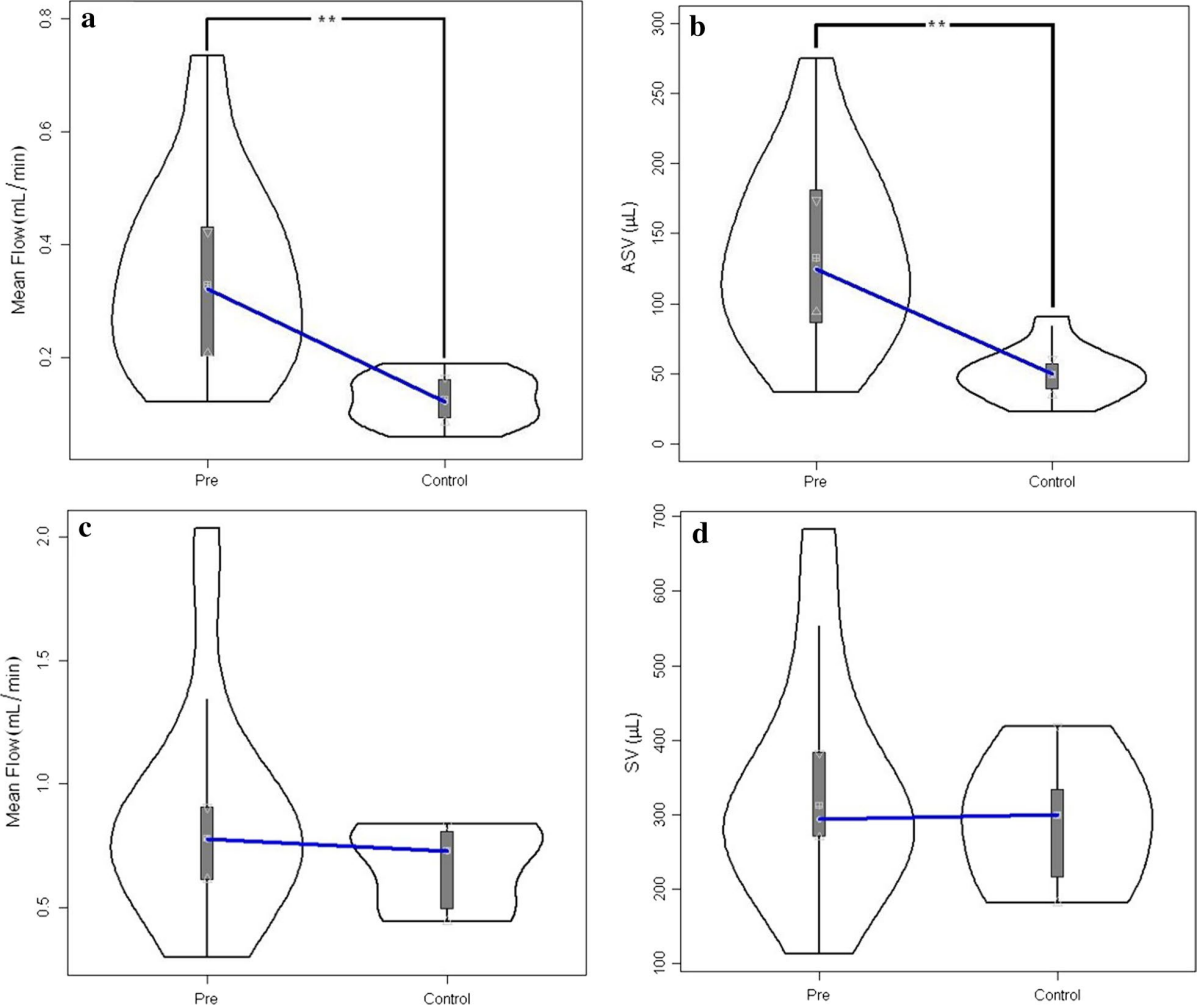
Violin plots for the comparison between the pre-treatment NPH group and controls: **a** Aqueductal caudal mean flow. **b** Aqueduct stroke volume. **c** Cisternal caudal mean flow. **d** Cisternal stroke volume. **p < 0.001

In the prepontine cistern, no hydrodynamic metrics were found to be significantly different between the pre-treatment group and the healthy controls (Table 1); however, the range of values was wider in the NPH group. Violin plots for the caudal mean flow and stroke volume for both the aqueduct and cistern are shown in Fig. 2 (cranial mean flow results are given in Table 1 but not plotted).

### Impact of shunt treatment on hydrodynamic metrics

**Fig. 3:**
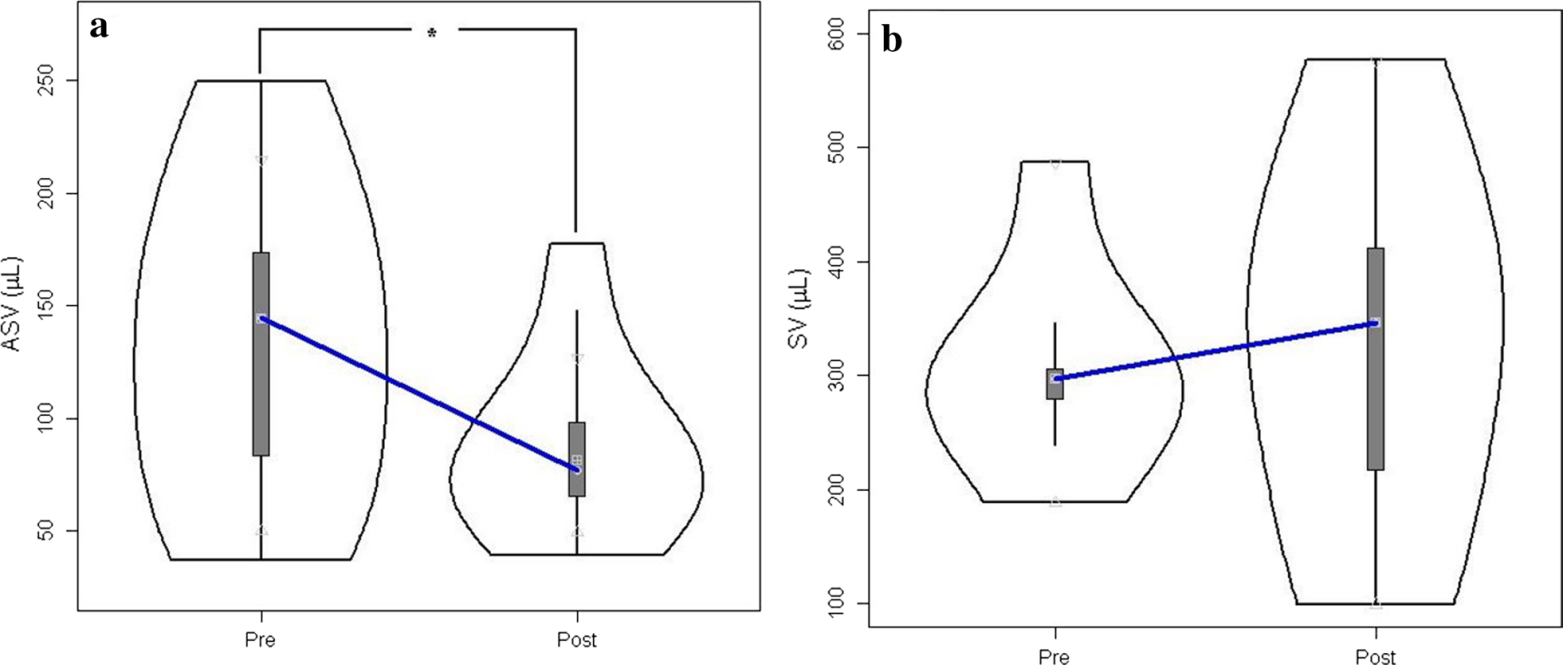
Comparison of pre-treatment and post-treatment stroke volume in **a** aqueduct and **b** prepontine cistern. The decrease in aqueduct stroke volume was significant *p < 0.05

Of the 12 post-treatment patients, three underwent VA shunt placement and nine were treated with VP shunt. In the cerebral aqueduct (nine matched pre-post pairs) ROI, caudal and cranial mean flow, and SV (Fig. 3a) were significantly reduced after shunt placement (Table 2). In the prepontine cistern (six matched pre-post pairs), there were no significant changes in velocity, flow rates, or SV (Fig. 3b). Complete results for both the aqueduct and cistern are shown in Table 2.

**Table 2:** The quantitative results from the pre- and post-shunt NPH groups for both the aqueduct and cistern

	Area (mm^2^)	Caudal max velocity (cm/s)	Cranial max velocity (cm/s)	Caudal mean flow (mL/min)	Cranial mean flow (mL/min)	SV (μL)
Aqueduct						
Pre-shunt (n = 9)	8.2 [3.3]* (5.1–16.4)	12.5 [9.7] (5.3–21.4)	9.5 [5.6] (4.5–13.0)	0.42 [0.33]** (0.12–0.70)	0.31 [0.23]** (0.09–0.46)	144.6 [127.6]** (37.1–249.6)
Post-shunt (n = 9)	7.8 [4.3] (3.1–9.0)	10.9 [4.5] (8.4–15.0)	6.6 [2.6] (5.4–11.9)	0.19 [0.17] (0.10–0.36)	0.15 [0.13] (0.09–0.39)	76.8 [55.1] (39.4–177.6)
Cistern						
Pre-shunt (n = 6)	58.0 [80.1] (45.2–255.7)	5.4 [4.1] (2.7–9.9)	4.7 [2.1] (3.0–9.3)	0.83 [0.30] (0.45–1.11)	0.41 [0.31] (0.29–0.89)	297.4 [99.4] (188.3–486.5)
Post-shunt (n = 6)	54.3 [78.7] (36.6–161.1)	5.1 [4.8] (1.9–11.9)	4.7 [5.2] (1.9–14.0)	0.82 [0.64] (0.2–1.29)	0.46 [0.41] (0.15–0.97)	346.5 [309.3] (99.3–576.0)

Significant differences denoted by *(p < 0.05) and **(p < 0.01) from the paired Wilcoxon signed rank. For each metric the median [iqr] are shown

### Peak flow latency

The PFL required ECG gating of the PC-MRI data and therefore a subset of the overall data was analyzed with the initial time point equal to the ECG signal performed in the MRI. For the cerebral aqueduct, 16 pre-treatment NPH patients and seven control patients were analyzed. The pre-treatment group showed a latency of 32.7% ± 8.16% compared to the control group latency of 34.4% ± 13.0% but the difference was not significant. In the prepontine cistern there was a significantly shorter PFL (p < 0.01) in the 15 pre-treatment patients (24.5% ± 6.3%) verse the five control subjects (29.6% ± 13.2%).

Following the shunt treatment, there were trends in both the cerebral aqueduct and prepontine cistern PFL toward control values; however, they failed to reach significance. In the cerebral aqueduct seven post treatment NPH patients had a mean ± SEM of 33.2 ± 12.5%. The post-treatment NPH patients had a slightly longer PFL of 27.9 ± 9.3% in nine patients, which again was not significantly longer than the pre-treatment group but trended toward the control group.

### Ventricle volume

The ventricle volume reported is the superposition of the lateral and third ventricles of the nine patients with pre- and post- treatment scans. The pre-shunt ventricular volumes ranged from 63.8 to 147.4 mL, mean and SEM 109.8 ± 8.2 mL. Following shunting, ventricular volumes were significantly reduced (p < 0.001), mean and SEM 91.51 ± 9.8 mL. Ventricle volumes were not compared for the control group.

### Stroke volume ratio

The stroke volume ratio was derived from the ratio of the ASV and the prepontine cistern SV for the pre-shunt, post-shunt, and control cohorts (Tables 1 and 2). For the pre-shunt group, there were 17 patients with technically adequate aqueduct and cisternal values resulting in a stroke volume ratio of 50.0 ± 7.3% mean and SEM, respectively. For the post-shunt patients, the stroke volume ratio was reduced but not significantly to 29.5 ± 7.2% (n = 7). The control group had a stroke volume ratio that was significantly lower (p = 0.0086) than the pre-shunt group, 17.7 ± 2.5%. The post-shunt and control groups did not differ significantly. In addition to the stroke volume ratio calculations for the entire pre- and post-shunt groups, the ratios for the six matched pre-post patients were also calculated. For the six matched patients, there was a significant reduction (p = 0.0321) from 50.2 ± 13.3% to 31.5 ± 8.3%.

### Patient outcome

Of the nine patients receiving pre- and post- treatment scans, eight received a VP shunt and one received a VA shunt. Of these nine patients, only one (a VP shunt) failed to clinically improve at the 6-month follow-up period.

## Discussion

In our study we investigated both cerebral hydrodynamic and peak flow latency (PFL) parameters in three groups, pre-treatment NPH patients, post-shunt NPH shunts, and controls within the cerebral aqueduct and the prepontine cistern. No significant differences were found in the mean CSF volumetric data for the prepontine cistern between the pre-treatment NPH group and the control group, although the range of values was higher in the pre-shunt NPH group. We documented prepontine SV values in NPH patients nearly twofold lower than the smallest value obtained in the control group. Balédent et al. reported that the prepontine cisternal CSF flow in patients with CH was smaller than healthy controls, but no quantities were given [17]. In a study published by Greitz, they reported SVs for the prepontine cistern in two healthy controls (SV = 0.33 ± 0.08 mL) and one CH patient (SV = 0.14 mL) [29]. It is difficult to make the comparisons between these results and those of other studies due to differences in ROI segmentation and imaging metrics.

Our study, like several others, demonstrated a significant difference in ASV and other hydrodynamic metrics between hydrocephalic patients and healthy controls [12–14, 23]. Balédent et al. implemented an automated method for segmentation of CSF and blood flow and found significant differences between area and SV within the aqueduct between healthy controls and patients with CH. Their results, based on 16 phase segments showed an increased area (17.0 mm^2^ vs. 8.0 mm^2^) and increased ASV (196.0 μL/mL vs. 51.0 μL/mL) for hydrocephalic patients versus healthy controls, respectively [17]. Furthermore, significant differences between CH (various etiologies) and healthy controls were also found by Abbey et al. within the aqueduct for area (10.0 ± 8.9 mm^2^, 2.0–27.0 mm^2^ and 2.0 ± 1.0 mm^2^, 1.0–4.0 mm^2^) and ASV (5.6–256.4 μL, 87.20 ± 79.04 μL and 1.9–33.2 μL, 17.4 ± 10.1 μL). However, differences in peak systolic and diastolic velocities were not found to be significant between the two groups [16], as we also found in our study. Ringstad et al. assessed net ASV and CSF aqueductal flow rate derived from PC MRI in patients with idiopathic NPH before and after ventriculoperitoneal shunt surgery. Net ASV was negative in 16 (76%) of 21 patients before shunt placement and in 5 (42%) of 12 patients after shunt placement, and increased from a median of − 5 μL (range − 175 to 27 μL) to a median of 1 μL (range − 61 to 30 μL; p = 0.04) [30].

Not unexpectedly, an increase in the mean ASV combined with no difference in the prepontine SV value resulted in an increase in the stroke volume ratio. Wagshul et al. investigated the CSF stroke volume ratio between the aqueduct and foramen magnum in 15 healthy adults [20]; although the study did not include CH patients they were able to define values for the stroke volume ratio in controls. In a related study by Balédent et al., the CH patients showed a significantly increased stroke volume ratio as compared to healthy controls, 42% and 11% respectively [17].

### Impact of shunting

Shunting remains the primary treatment of NPH; however, there remains controversy over the selection of those patients who are likely to respond to shunt. Although not investigated in this work, CSF flow quantification with MRI [12, 14, 31, 32] has been used along with other methods such as radionuclide cisternography [33], overnight ICP monitoring [34–41], CSF tap test [42–44], extended lumbar drainage (ELD) [40, 45, 46], and CSF infusion (outflow resistance [42, 47–54]) to aid in the diagnostic/prognostic assessment of these patients. Following shunt surgery, cisternal CSF flow and SV were slightly increased but did not reach significance. Similar results have been published supporting that CSF pulsatility and stroke volume through the aqueduct is correlated with a positive response to shunting in patients with NPH [55].

The diversion of CSF resulted in a significant decrease in both the caudal and cranial mean flow (p < 0.05) and ASV (p < 0.05) in the aqueduct, which is consistent with one similar study [16]; however, in that study, peak velocities (caudal and cranial) and area were not found to be significantly reduced [16]. Again, there have been several studies that show a decrease in the ASV, flow, and velocity following a shunt procedure [13, 16, 17, 32]; although the mechanisms underlying this decrease in hydrodynamics have been relatively unexplored.

The CSF shunts used in our study include a valve mechanism that is a one-way check valve that has a pre-set opening pressure. For the post-shunt studies, we assume that the system is in steady-state, and therefore CSF flow down the shunt would occur when the peak CSF pulsatile pressure exceeds the threshold value, resulting in microbolus flow. In a study by Miyaje et al., the CSF flow through the shunt was measured using a microflowmeter in seven NPH patients; which included changes in valve opening pressure and changes in posture (sitting and standing) [56]. For patients in the recumbent position (same as the MRI), the study reported that, at low valve opening pressures, flow within the shunt varied between 100 and 200 μL/min. In our study, there was an average decrease in the caudal and cranial mean flow of 147.2 ± 105.9 and 93.0 ± 33.3 μL/min, respectively (median data reported in Table 2). The magnitude decrease in mean flow volume through the aqueduct is approximately equal to the data reported by Miyaje et al. for the flow through a shunt while in the supine position.

The stroke volume ratio following shunt surgery showed a decrease in the larger (unmatched) cohort but was not significant; however, in the six matched patients with pre and post scans, there was a significant reduction in stroke volume ratio following the surgery (p = 0.0321). Furthermore, the pre-shunt group had a significantly higher stroke volume ratio than the control group (p = 0.0086) which correlated well with Balédent’s work described above. The absolute value of the ratios cannot be directly compared to work by Balédent et al. or Wagshul et al. because of the difference in location for the SAS stroke volume measurement. When investigating the contributions of the aqueduct and the cistern, the significant differences shown would be expected. Although the significant decrease in stroke volume ratio seems to be driven by the significant decrease in ASV shown in Table 1 and Table 2, the upward trend of cisternal SV following surgery could support the hypothesis of redistribution of intracranial CSF pulsations; however, additional work is needed to confirm or reject the stated hypothesis.

### Peak flow latency

**Fig. 4:**
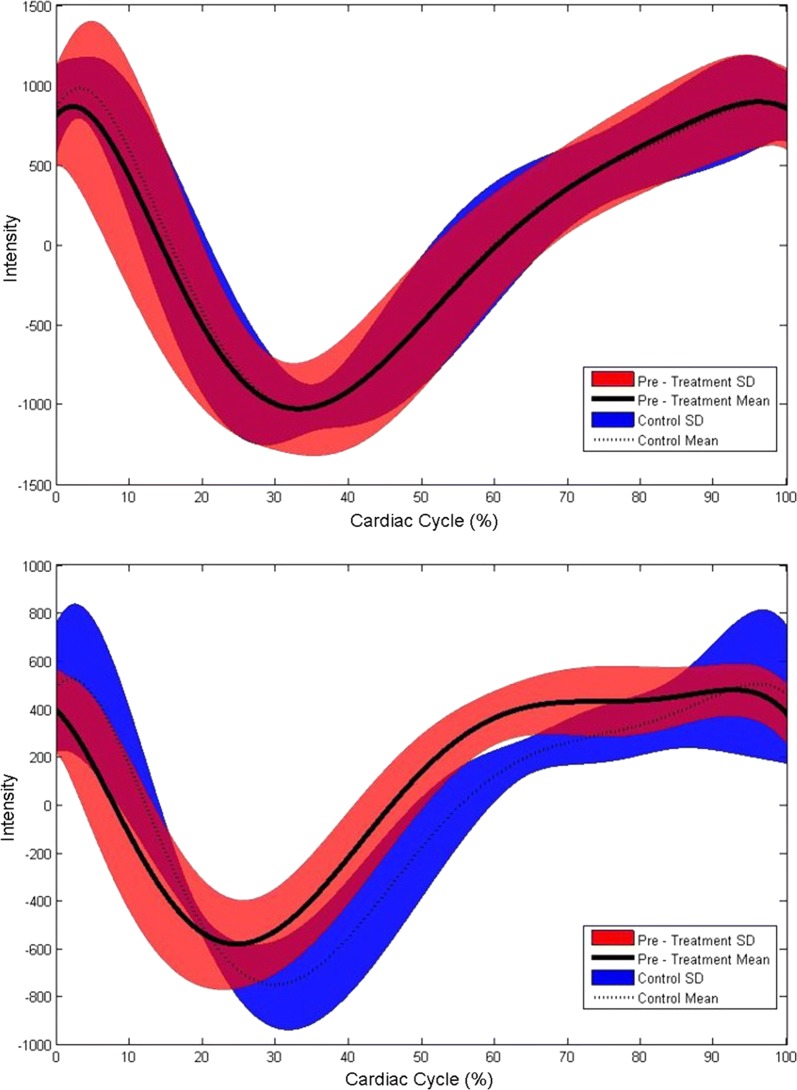
Mean uncalibrated flow curves (voxel intensity) over the cardiac cycle. Top) aqueduct and bottom) prepontine cistern for the pre-treatment and control groups. The curves are the average of the polynomial fit (6th degree) of the entire group (SD also shown as shaded region). The difference seen between the peak latency (defined as the minimum point of the curve) is significantly shorter (p < 0.01) in the pre-treatment group than in the control group for the prepontine cistern. The objective of this figure is to show the phase change during the cardiac cycle

To supplement the volumetric analysis, latency metrics were also investigated in this study. Unlike the volumetric results, the aqueduct showed no significant differences in PFL between the groups; however, there was a trend showing a shortened latency in the pre-treatment group compared with the healthy controls. In the prepontine cistern the pre-treatment group showed a significantly shorter PFL compared with the healthy controls (Fig. 4). This change in CSF latency partially supports our hypothesis that pre-treatment NPH patients should have reduced latency in both the cerebral aqueduct and prepontine cistern. Although we were not able to show a difference in aqueductal latency between the two groups, the change in cisternal latency is an interesting finding as it supports the work from Egnor’s model of CH of redistribution of CSF pulsations in the cranial vault.

The PFL calculations were dependent on two variables: the degree of the polynomial used to increase the temporal resolution and the percentage of voxels removed from the original ROI. Table 3 shows the corresponding p-values for the prepontine cistern for a number of different combinations of degree of polynomial (4–10) and percentage of voxels removed. The analysis reported is for a six-degree polynomial and 75% voxel removal (marked with an asterisk in Table 3). There is minimal impact on the overall significance of the PFL by altering these two variables between the pre-treatment NPH group and healthy controls. However, when 95% of the data is removed (thus a majority of the voxels) and the degree of the polynomial fit is relatively high (8–10) the results are no longer significant. This trend is expected; as voxels are removed there is more influence from individual voxels, increasing the noise in the results along with “over-fitting” from the high degree polynomial fit. Taken at both extremes, a poor-fitting or over-fitting polynomial will confound the final results. Finally, when no polynomial fit is performed the results become very irregular and significance is rarely reached (Table 3).

**Table 3:** p-value from the Mann–Whitney Rank sum comparing pre-treatment NPH and control patients for prepontine cistern peak flow latency

	Percentage of low-correlated voxels removed from the ROI						
Degree of fit	1%	10%	25%	50%	75%	90%	95%
No poly fit	0.076	0.076	0.061	0.107	0.040	0.094	0.061
4	0.007	0.009	0.009	0.009	0.009	0.007	0.004
5	0.009	0.009	0.011	0.009	0.011	0.007	0.015
6	0.009	0.007	0.009	0.009	0.009*	0.007	0.015
7	0.023	0.014	0.014	0.025	0.023	0.009	0.036
8	0.044	0.036	0.044	0.044	0.029	0.009	0.067
9	0.036	0.036	0.044	0.040	0.029	0.011	0.067
10	0.066	0.055	0.036	0.044	0.036	0.011	0.116

For the analysis a six degree polynomial was used to fit the flow data and the top 25% of the voxels were used in the calculation (represents 75% of the low-correlated voxels being removed) p value shown with an *.The “No Poly Fit” row contains the results excluding any polynomial fitting

As established earlier, the pathophysiology of NPH has been discussed to great extent in the literature and one can find a wide variety of possible root causes. One topic that has been relatively unexplored is the role of parenchymal changes leading to pathogenesis and symptoms in NPH. The variability in shunting success and neurodegenerative pathology in some patients may indicate that NPH is not quite as simple as misguided CSF, and that the pathology may lie in parenchymal abnormalities [57]. A new technology known as magnetic resonance elastography (MRE) [58], has the ability to quantify the mechanical properties of the microstructure of the parenchyma. Using this technology, a study by Freimann et al. investigated the changes in mechanical properties of the pre- and post- shunt brain of NPH patients and compared those changes to healthy controls [59]. Two significant findings were reported: first, there was a significant difference in shear elasticity (μ) between the control group and the pre-shunt NPH group that did not correct following shunting. Shear elasticity is a measure of global brain stiffness; therefore, the significant decrease in μ represents a decrease in brain stiffness of the NPH patients. The other finding showed a significant decrease in a parameter known as the connectivity parameter (α) between the healthy control group and the pre-treatment NPH group. Following surgery, the connectivity parameter returned to normal ranges. Unlike the brain stiffness the connectivity parameters is slightly more abstract, being described by the authors as being “sensitive to the geometry of the mechanical network” [59]. Succinctly, there is a reorganization of the parenchymal microstructure toward healthy values (more organized). We hypothesize that this reorganization following shunting could contribute to the reversal in both the volumetric and latency trends in the prepontine cistern. As previously discussed, the aqueductal change is also influenced by the removal of the CSF via shunting.

### Potential study pitfalls

Our study was limited by the number of subjects, particularly patients who were studied both pre- and post-shunt, as well as the control group. Furthermore, the lack of specific age and ventricular volume matching also was also a limitation. There were also significant technical challenges. The prepontine cistern is a complex anatomical structure that includes the basilar artery as well as small veins. Arachnoidal septations within the cistern, if present, could possibly direct pulsatile CSF in directions not aligned with the axis of the brainstem (Fig. 1). Each or both of these could have contributed to errors in the automated segmentation algorithm, resulting in both inaccurate ROI areas and flow values. Ultra-high resolution imaging with stronger Tesla MRIs and multiplane imaging interpreted with mathematical modeling could address these gaps in information regarding CSF dynamics as a discovery and exploratory tool [60], but were not possible here. Additionally, technical challenges in latency calculations using the percentage of cardiac cycle could also introduce some level of variability. Future studies should investigate absolute time to peak-systolic flow.

## Conclusion

For our purposes, PC-MRI provided a method to quantify the hydrodynamic changes that occur following a CSF diversion. Furthermore, we were able to compare those hydrodynamic changes with previously reported values for CSF flow within a shunt. Although several studies have quantified the changes in aqueduct flow between groups and a few studies have investigated prepontine cistern flow, our study is the first to investigate both regions for NPH pre- and post- treatment. Following shunt treatment, the aqueductal CSF metrics decreased toward control values. This is contrary to the prepontine cistern metrics that trended upwards (although not significantly) away from the normal values established in this study. Additionally, our study is the first to report latency differences within the prepontine cistern CSF flow between healthy controls and pre-treatment NPH patients.

## Abbreviations

CSF: cerebrospinal fluid
PC-MRI: phase-contrast MRI
NPH: normal pressure hydrocephalus
PFL: peak flow latency
ASV: aqueductal stroke volume
ROI: region of interest
SV: stroke volume
VP: ventriculoperitoneal
VA: ventriculoatrial
ETV: endoscopic third ventriculostomy
CBF: cerebral blood flow
